# The “sausage” abscess: abscess of the liagamentum teres hepatis

**DOI:** 10.1259/bjrcr.20150139

**Published:** 2016-11-02

**Authors:** Debraj Sen, Vijinder Arora, Ravdeep Singh Sohal, Pothina Sree Hari

**Affiliations:** ^1^Department of Radiology, Military Hospital Jodhpur, Jodhpur, India; ^2^Department of Radiology, Sri Guru Ramdas Institute of Medical Sciences and Research, Amritsar, India; ^3^Department of Surgery, Military Hospital (CTC), Pune, India; ^4^Department of Surgery, Army Hospital (R&R), New Delhi, India

## Abstract

An abscess of the ligamentum teres hepatis is a very rare cause of acute abdomen and can present a diagnostic dilemma. A 40-year-old diabetic male presented with obstructive jaundice and cholangitis. An ill-defined, sausage-shaped, tender parasagittal supraumbilical mass was palpable on the right side. Murphy’s sign was negative. Laboratory investigations revealed polymorphonuclear leukocytosis (total leukocyte count 19,000 mm^–3^), elevated alkaline phosphatase (400 IU l^–1^), conjugated hyperbilirubinaemia (16 mg dl^–1^) and elevated blood glucose (240 mg dl^–1^). Ultrasonography and MR cholangiopancreatography revealed cholecystolithiasis, obstructive choledocholithiasis, abscess of the ligamentum teres hepatis and left portal thrombosis. Under ultrasound guidance, pus was aspirated from the abscess and the patient was started on broad-spectrum intravenous antibiotics, insulin and low-molecular-weight heparin. He subsequently underwent endoscopic retrograde cholangiopancreatography with sphincterotomy and stone extraction. On the tenth day post admission, he underwent laparoscopic cholecystectomy and excision of the ligament. The patient made an uneventful recovery and was discharged on the seventh post-operative day. On follow-up, the patient remained asymptomatic with normal biochemical parameters. This article highlights the importance of suspecting and identifying an abscess of the ligamentum teres hepatis when a patient with acute abdomen presents with a sausage-shaped right parasagittal mass, especially in the setting of cholangitis, cholecystitis or omphalitis.

## Summary

Localized accumulations of pus or abscesses are ubiquitous. However, an abscess of the ligamentum teres hepatis or round ligament (remnant of the obliterated fetal left umbilical vein) is a very rare occurrence. Only about 16 cases in adults and 6 cases in neonates and infants have been reported until 2016. An abscess at this location presents as an acute abdomen and can be perplexing. It may be erroneously diagnosed as an abdominal wall abscess and managed non-surgically, which is usually unsuccessful. Hence, familiarity with this entity and its early recognition is essential for the radiologist, surgeon and paediatrician. This article presents a case of an abscess of the liagamentum teres hepatis associated with left portal thrombosis consequent to cholecystolithiasis, obstructive choledocholithiasis and cholangitis. The purported aetiopathogenesis, imaging findings and management of this condition are also discussed.

## Case report

A 40-year-old male, suffering from diabetes mellitus for the past 2 years and on irregular treatment, was brought in with a history of progressive jaundice of 10 days’ duration, and fever and right upper abdominal pain for the past 3 days. There was also a history of passage of clay-coloured stools. The patient mentioned occasional post-prandial self-limiting right upper quadrant pain in the past, for which he did not get himself investigated. On examination, he was found to be febrile with a temperature of 102 °F and appeared dehydrated. His pulse was 100 min^−1^, regular but low in volume, with a blood pressure of 110/70 mmHg and respiratory rate of 18 min^−1^. He had marked icterus. Murphy’s sign was negative. An ill-defined, sausage-shaped, tender parasagittal supraumbilical mass was palpable on the right side. Erythema was noted on the skin around the umbilicus; however, there was no umbilical discharge. Laboratory investigations revealed polymorphonuclear leukocytosis (total leukocyte count 19,000 mm^–3^ ), with elevated liver enzymes (alkaline phosphatase 400 IU l^−l^, alanine transaminase 90 IU l^−l^, aspartate transaminase 100 IU l^−l^) and conjugated hyperbilirubinaemia (16 mg dl^−1^). His blood glucose was 250 mg dl^−1^. The rest of the biochemical tests were normal.

**Figure 1.**
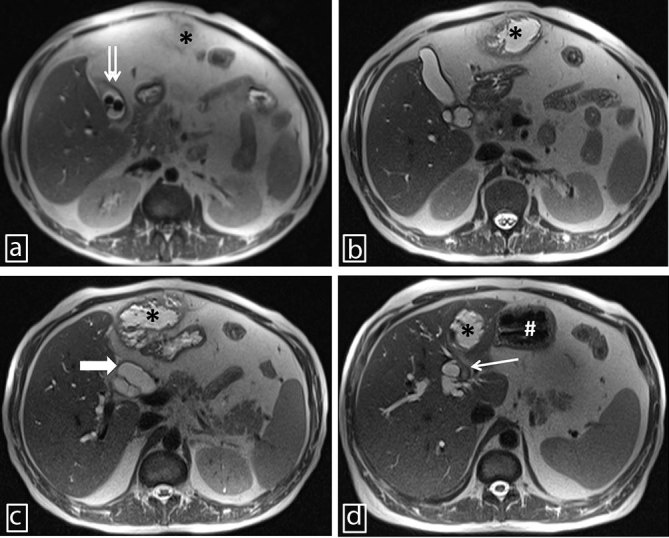
A panel of axial half-Fourier single-shot turbo spin-echo *T*_2_ images (caudal to cranial; a–d) showing cholecystolithiasis (double arrows), abscess of the ligamentum teres hepatis (asterisks), dilated common bile duct (thick arrow), stomach (hash) and thrombosed left branch of the portal vein (thin arrow).

**Figure 2.**
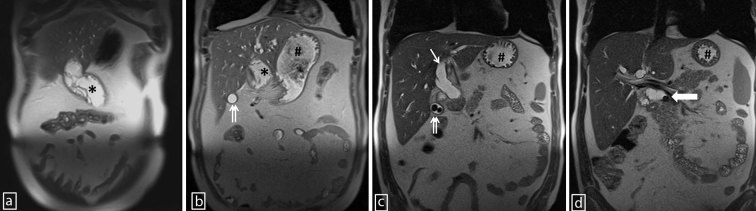
A panel of coronal half-Fourier single-shot turbo spin-echo *T*_2_ images (anterior to posterior; a–d) depicting abscess of the ligamentum teres hepatis (asterisks), cholecystolithiasis (double arrows), stomach (hashs), dilated left main hepatic duct (thin arrow) and common bile duct calculus (thick arrow).

**Figure 3.**
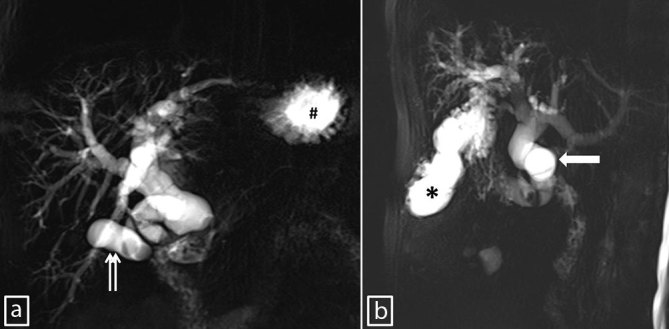
A panel of anteroposterior (a) and oblique sagittal (b) single-shot fast-spin-echo maximum intensity projection MR cholangiopancreatography images showing the dilated biliary tree, gallbladder with intraluminal filling defects (cholecystolithiasis; doublearrows), stomach (hash), dilated common bile duct (thick arrow) with a filling defect (choledocholithiasis) at its midsegment and abscess of the ligamentum teres hepatis (asterisk).

Transabdominal ultrasonography of the abdomen revealed cholecystolithiasis. However, there were no signs of cholecystitis. Bilobar dilatation of the intrahepatic biliary radicles was noted and the common bile duct measured 20.0 mm in diameter. A calculus measuring 8.2 mm was seen in the middle segment of the common bile duct. A tubular cystic structure with echogenic debris was visualized, extending from the umbilicus off midline on the right side to the inferior surface of the medial segment of the left lobe of the liver. A subsequent MR cholangiopancreatography (Symphony Tim 1.5 T MRI scanner, Erlangen, Germany) also showed cholecystolithiasis and choledocholithiasis with an 8.0 mm-sized calculus in the distal part of the supraduodenal common bile duct. The entire biliary tree proximal to the calculus was dilated. A sausage-shaped cystic lesion with thick shaggy walls was seen on the right side, extending from the umbilicus cranially up to the umbilical fissure. This lesion measured 16.0 × 3.5 × 3.5 cm (craniocaudal × anteroposterior × transverse). The normal flow void in the left branch of the portal vein was replaced by hyperintense signal intensity (Figures 1–3). These findings were consistent with cholecystolithiasis, obstructive choledocholithiasis with cholangitis, left portal vein thrombosis and an abscess of the ligamentum teres hepatis.

Under ultrasound guidance, 80 ml of pus (which later grew *Escherichia coli* on culture) was aspirated from the abscess on the day of admission and the patient was started on broad-spectrum intravenous antibiotics and insulin. He was also started on injectable low-molecular-weight heparin. The next day, he underwent endoscopic retrograde cholangiopancreatography with sphincterotomy and stone extraction. The patient gradually recovered with subsidence of fever and improvement of the clinical parameters. On the tenth day post admission, he underwent laparoscopic cholecystectomy and excision of the residual abscess cavity. The patient made steady recovery after the surgery and was discharged on the seventh post-operative day. Ultrasonography performed on the day of discharge was normal except for evidence of cholecystectomy. Colour Doppler flow imaging revealed normal colour flow in the left portal vein. On follow-up, the patient remained asymptomatic with well-controlled blood sugar and normal liver function.

## Discussion

The diaphragmatic peritoneal reflections on the liver surface are referred to as coronary ligaments and their lateral reflections on either side are called the right and left triangular ligaments, respectively. Anteriorly, in the centre, these reflections form the falciform ligament, which extends as a thin membrane connecting the liver surface to the diaphragm, abdominal wall and umbilicus. The ligamentum teres hepatis (round ligament of the liver), which is the obliterated left umbilical vein, measures approximately 17 cm in length and runs along the inferior margin of the falciform ligament from the umbilicus to the umbilical fissure (between the left and quadrate lobe) on the inferior surface of the liver.^1,2^

An abscess of the ligamentum teres hepatis or round ligament is a very rare occurrence. Only about 16 cases in adults and 6 cases in neonates and infants have been reported until 2016. Most cases in adults have been reported in the elderly population.

The reported causes of abscess of the ligamentum teres hepatis are acute calculous cholecystitis, cholangitis and pancreatitis in adults, and omphalitis and infected ventriculoperitoneal shunt in children.^3–10^ Lipinski et al^11^ proposed contiguous spread of infection from omphalitis *via* the round ligament as the cause of this abscess in infants. The paraumbilical veins connect the superficial periumbilical venous network to the left portal vein to form a portal–systemic venous anastomosis. This venous network might explain the mechanism of spread of infection from omphalitis into the falciform ligament in the absence of a round ligament (post-operative cases).^7^ The cholecystic veins drain directly into the portal vein and the pericholedochal venous system. It is likely that the infection spreads from the biliary tree *via* the paraumbilical veins to the falciform ligament, leading to abscess formation.^5^ Lymphatic spread of infection has also been postulated as a cause of abscess formation.^3^

The major causes of portal vein thrombosis are liver cirrhosis, neoplasms and haematological malignancies, infection and pancreatitis. In the absence of cirrhosis or neoplastic disease, approximately 10–25% cases of portal vein thrombosis are associated with sepsis. Portal pyaemia and thrombosis may occur secondary to appendicitis, biliary tract infection, post-abdominal surgery sepsis, amoebic colitis, acute necrotizing pancreatitis and diverticulitis.^4^ In our patient, cholangitis was likely to have caused portal pyaemia, portal vein thrombosis and spread of infection to the ligamentum teres hepatis.

Potential complications of missed or delayed diagnosis of an abscess of the ligamentum teres hepatis are peritonitis, sepsis, mass effect causing extrinsic intestinal obstruction and portal thrombosis.

Management of ligamentum teres abscess involves use of broad-spectrum intravenous antibiotics to cover the gastrointestinal flora and elimination of the abdominal focus of infection. Aspiration of pus followed by surgical excision of the abscess should be performed immediately on diagnosis if features of peritonitis are present, owing to the risk of extensive spread and severe peritonitis.^4^ In other cases, a more delayed approach may be followed, as in the case of our patient. Antibiotic therapy and surgical excision are the mainstay of treatment; however, conservative management with endoscopic biliary drainage and antibiotics has also been successful.^3–5,9^ Any predisposing condition such as acute calculous cholecystitis, cholangitis, choledocholithiasis and omphalitis will also need to be simultaneously addressed.

## Conclusions

An abscess of the ligamentum teres hepatis is a very rare cause of acute abdomen that can present a diagnostic dilemma. Intra-abdominal infections leading to portal pyaemia and consequent spread of infection *via* the paraumbilical veins to the falciform ligament can lead to abscess formation. This entity should be suspected when a patient with acute abdomen presents with an elongated supraumbilical right parasagittal mass, especially in the setting of cholangitis, cholecystitis or omphalitis.

## Learning points

1. Abscess of the ligamentum teres hepatis is a rare cause of acute abdomen.
2. It should be suspected when a sausage-shaped, right parasagittal supraumbilical mass is palpable in the setting of cholangitis, cholecystitis or omphalitis.
3. It occurs owing to portal pyaemia and consequent spread of infection *via* the paraumbilical veins to the falciform ligament.
4. It may erroneously be diagnosed as an abdominal wall abscess. The diagnosis is confirmed by ultrasonography and/or CT or MRI.
5. Complications include peritonitis, portal thrombosis, sepsis and mass effect, causing extrinsic intestinal obstruction.
6. Scrupulous umbilical cord hygiene is essential in neonates to prevent omphalitis and secondary abscess formation.
7. Management of the abscess is three-pronged: broad-spectrum intravenous antibiotics to cover the gastrointestinal flora, elimination of the inciting focus of infection (such as calculous cholecystitis, choledocholithiasis, etc.) and drainage of pus.

## Consent

Written informed consent was obtained from the patient to publish this case. All patient images have been anonymized and the patient is not identifiable from the images/medical information included in the article.
